# Patient oriented research in mental health: matching laboratory to life and beyond in Canada

**DOI:** 10.1186/s40900-021-00266-1

**Published:** 2021-04-26

**Authors:** Jenessa N. Johnston, Lisa Ridgway, Sarah Cary-Barnard, Josh Allen, Carla L. Sanchez-Lafuente, Brady Reive, Lisa E. Kalynchuk, Hector J. Caruncho

**Affiliations:** 1 Division of Medical Sciences, University of Victoria, Victoria, BC Canada; 2 Patient Partner, BC SUPPORT Unit, Victoria, BC Canada; 3 Patient Partner, Victoria, BC Canada

**Keywords:** Patient-oriented research, Mental health, Laboratory preclinical research, Patient partners, Knowledge transfer

## Abstract

As patient-oriented research gains popularity in clinical research, the lack of patient input in foundational science grows more evident. Research has shown great utility in active partnerships between patient partners and scientists, yet many researchers are still hesitant about listening to the voices of those with lived experience guide and shape their experiments. Mental health has been a leading area for patient movements such as survivor-led research, however the stigma experienced by these patients creates difficulties not present in other health disciplines. The emergence of COVID-19 has also created unique circumstances that need to be addressed. Through this lens, we have taken experiences from our patient partners, students, and primary investigator to create recommendations for the better facilitation of patient-oriented research in foundational science in Canada. With these guidelines, from initial recruitment and leading to sustaining meaningful partnerships, we hope to encourage other researchers that patient-oriented research is necessary for the future of mental health research and foundational science.

## Plain English summary

This paper presents a roadmap for patient-oriented research in mental health in Canada. At the level of laboratory science, the direct involvement of patients and their collaboration with researchers on experimental development and dissemination of results has been scarce. With voices of collaborative patient partners, graduate students and the principal-investigator, it makes a clear case for involving people with lived experience within translational mental health research, which has shown positive outcomes that could potentially shape public policy and future practice. Starting with the premise that meaningful engagement spurs meaningful results, the paper addresses current literature, a discussion of the challenges faced by researchers and patient partners and includes a consideration of COVID-19 consequences. Ending with clear recommendations, this paper is an exciting call to action for involving patient partners in the full research cycle of mental health research and beyond.

## Background

### Patient-oriented research

Patient-oriented research (POR) endorses the prioritization of an active partnership between multiple stakeholders (e.g. patients, clinicians, researchers etc.) in order to promote improvement of the current healthcare system. While incorporating patient perspectives into the healthcare system seems like it should be obvious, the current evidence-based model and clinical practice guidelines in Canada leave little room for input from people with lived experience (PWLE) [1, 2].

Within the last two decades these opinions have shifted, with a general increase in empowerment of patients and democratization of science largely in part to patient group lobbying [3]. Certain countries, such as the United Kingdom (UK) established methods of patient involvement and user-led research earlier, through the National Institute of Health Research (NIHR) UK standards for public involvement. These six standards provide a comprehensive framework to foster relationships between health care workers and the public, promoting involvement at multiple levels of research [4]. In Canada, this manifested later through the Strategy for Patient-Oriented Research (SPOR) created by the Canadian Institute of Health Research (CIHR). A central tenet of this framework is that of patient engagement, which they define as “occurring when patients meaningfully and actively collaborate in the governance, priority setting, and conduct of research, as well as in summarizing, distributing, sharing, and applying its resulting knowledge” [5]. Reflecting the report released by the INVOLVE Advisory Group associated with the NIHR, this level of engagement would define patients as co-producers of the research described in our report [6]. On the IAP2 (International Association for Public Participation) spectrum, this would place the engagement between “Collaborate” and “Empower” [7]. The term patient partners will be used to describe those with active engagement who function as co-producers in our research, a term preferred by our PWLE collaborators.

The benefits of the change towards valuing patient input has been proven on a number of levels: that the experiential knowledge of patients increases the quality and relevance of health research, and that patient participation in research can lead to a better acceptance of research outcomes by others [8]. Patient partners, who have unique backgrounds and experiences also provide new perspectives for the co-production of research ideas and experimental avenues. This is not limited to their own experiences with mental health disorders. For example, a patient partner with mathematical expertise could mentor investigators on proper statistical use, or someone with graphic design experience could create materials for further recruitment. Some studies have attempted to quantify direct patient involvement in biomedical research outcomes by finding evidence of direct demands or concerns recorded from patients [9]. This quantification could be problematic in the attempt to frame patient-oriented research under the traditional medical model that has historically failed in including patient perspectives. In foundational laboratory-based science, there is a clear bias towards purely quantitative results which hinders scientific advancement by potentially disqualifying essential information. The benefits of qualitative evidence in science are clear from the fields of psychology, sociology, and more who have contributed great advances to the scientific community. The necessity to educate lab-based scientists to accept viewpoints that would be classified as qualitative is evident. Even with evidence of beneficial outcomes POR faces many challenges regarding proper incorporation into biomedical sciences. Gaining insight into the exclusion mechanisms that those with lived experience will encounter is a priority. These include behaviors (e.g. granting them less speaking time, attention, or respect) or verbal communications (e.g. clinicians sidelining issues they feel are not their responsibility, speaking in jargon) that make the patient partner feel uncomfortable and undervalued [3, 10]. A recent study [11] showed that many biomedical scientists and health care service workers still prioritized perceived expertise over experience, even with conscious positive attitudes towards patient and public involvement.

Systemic issues also discriminatorily impede the participation of various PWLE. Racial and ethnic disparities in health research – To our knowledge, there have been no studies that have evaluated racial discrimination in POR. However, there is clear evidence of systemic and individual discrimination in healthcare settings including quality of treatment, economic barriers, bias in clinical trial participation, lack of access to proper healthcare, and more [12–16]. There is a high probability that these issues would be present in POR as well. For example, research on attempts to rectify these disparities have found that patients feel more comfortable with clinicians of the same race and gender [14]. It would follow that the increased comfort would extend to the PWLE-researcher relationship in a laboratory setting. However, recent data from 88 Canadian Universities show a lack of representation of marginalized populations. Out of full-time faculty, 40.2% were women, 20.9% were non-white, 1.3% were indigenous, and 21.8% had diagnosed disabilities [17, 18]. No data was collected for LGBTQIA2S+ (Lesbian, Gay, Bisexual, Transgender, Queer and/or Questioning, Intersex, Asexual, Two-Spirit, plus the countless affirmative ways in which people choose to self-identify), or intersectionality of these groups. In STEM (science, technology, engineering, and mathematics) the “leaky pipeline” phenomenon contributes to greater marginalization. In the U.S., white women make up 23.4% of tenure-track faculty in STEM whereas women of colour only represent 2.3% [19]. If faculty diversity is not increased, the likelihood of PWLE interacting with equivalent counterparts is low, and contributes to participant discomfort. This is just one example of how marginalized groups could face greater challenges in participating and having their ideas heard in laboratory-based POR. Solutions for discrimination and increased barriers for different marginalized groups in Canadian POR needs extensive further research and detailed demographics still not available.

Our laboratory-based research focuses on the characterization of potential novel antidepressants and the study of biomarkers for different depression subtypes and predictions of treatment responsiveness [20, 21]. We are foundational researchers, who focus on the discovery and investigation of basic mechanisms in the human body. While we do not directly treat patients, we are in the unique position of working alongside PWLE to develop novel experimental avenues, as well as the development of unique methods to disseminate our research to the public. It is essential for foundational biomedical researchers to partner with patients to determine strategies in overcoming their biases and to develop a working framework that allows for true partnership and respect between patient partners and researchers.

### Translational research

There are evident gaps between foundational research, clinical sciences, and the public health setting. Each group uses their own jargon and specialized techniques that makes it difficult to bridge the gaps between them. Translational research aims to bridge these different fields aiming to foster joint efforts to better advance strategies and discoveries with practical health outcomes. The National Center for Advancing Translational Sciences defines this concept as “the process of turning observations in the laboratory, clinic, and community into interventions that improve the health of individuals and populations …” [22]. Translational science could also be described in a bidirectional broader sense, where experiences of individuals and populations are turned into directives for foundational and clinical research. Effective knowledge transfer is a key part of translational research, as communication between all parties cannot be facilitated until all groups are using the same language and terminology.

This has not always been successful in science, with the common “valleys of death” metaphor used to describe the information that is lost between each stage of research [23]. Failures of translation in biomedical sciences are evident in many situations, particularly in cases of translation from the community to science laboratories [24–26]. In fact, clinical practice guidelines can go against patient preferences, with clinicians rarely factoring in key aspects such as socioeconomic or work status into treatment options [1]. The lack of incorporation of community values has led to some proposing a patient emancipation movement, championing greater control over medical decisions, personal autonomy, knowledge, and respect from the greater healthcare system [10].

To incorporate PWLE into foundational research, there are multiple facets of knowledge transfer that demand improvement. The first is the adaptation of experiential data from those with lived experience to experiments that can be performed in the laboratory. In most foundational research, individual qualities are erased to control for extraneous variables, which is not a reflection of the reality for many PWLE. The erasure of individual variables contrasts with many of the questions posed by patients (e.g. “What is the right treatment for me?”, “How does socioeconomic status impact treatment availability?”, “What are the best treatments for different lifestyles?”). An example of a solution for this is Priority Setting Partnerships (PSP) that is described in further detail later in this report.

The second translational component of POR is the translation of foundational research to those with lived experience. As mentioned previously, knowledge is a powerful tool for patients to gain equal respect when negotiating the terms of their treatment or the focus of future research [3, 10, 12]. Aside from this, certain patient partners express their own interest in biological and foundational research, and participation in the laboratory setting could provide a learning experience. One of our patient partners has expressed interest in working with the Animal Care Unit that we use for our research. Patient partners are also eager to incorporate their own experiences into research, such as one of us who is a lawyer and has provided invaluable insight into administrative processes and furthering POR in our community. The final component, included in nearly all of the POR research thus far, is effectively translating the findings from the foundational scientists and PWLE to a larger audience that includes clinicians, other patients, and the wider community [26, 27]. This is considerably more difficult than it seems at first glance, with questions such as what knowledge, to whom, by whom, and how the information should be distributed [28, 29].

At each stage, identifying potential barriers to translation is necessary to move forward. For the initial collaboration between those with lived experience and foundational researchers, there would need to be equal footing on which the patients and researchers can stand. Another potential barrier is that of jargon typically used in the scientific community. All researchers should attempt to place their ideas in lay terminology, and that which is not possible should be provided to patient partners beforehand so that they may be familiarized with the terminology. All of these steps and potential challenges need to be considered when engaging in any form of medical research, however this is particularly important with POR in the laboratory setting.

An established method of engaging patients in research is by Priority Setting Partnerships (PSPs), which allow PWLE to set research priorities in certain health conditions. The James Lind Alliance (JLA) [30] has developed a framework for this research that has been successfully used in the UK, United States and Canada [31–39] for various conditions, including mental health disorders**.** However, in many articles patient engagement in the priority setting process was not conducted in a laboratory setting, an area with unique challenges. This paper aims to gather input provided by patients to update guidelines on how to create meaningful and engaging partnerships in POR in the laboratory setting. It is expected that these results will be particularly meaningful to patient partners in British Colombia and will provide a framework to expand our engagement in POR further across the province.

### POR in mental health research

Those with lived experience of mental health disorders face many unique challenges in comparison to other groups of patients. Before beginning this section, we would like to acknowledge that those diagnosed with mental health disorders encompass a wide range of people, that can include clinicians and researchers. In reality, there are no boundaries between “patient”, “clinician”, and “researcher”. While we use these labels separately, please keep in mind the overlap and potential stigma experienced by those who fit into multiple categories.

Those diagnosed with mental health disorders are often viewed as less competent to make decisions regarding their care, a perceptual bias that does not account for fluctuating cycles of wellbeing or large differences in disorder severity. Unfortunately, this perceptual bias and systemic discrimination poses a challenge when implementing a model whereby a partnership between the patient and researcher is more egalitarian. There is also some risk related to the capacity of the person to participate in this form of research, and a discussion on roles and responsibility must be personalized to the individual patient. In these cases, the inclusion of caregivers and family members in the decision-making process is valuable, as demonstrated with shared decision-making with acutely ill in-patients with schizophrenia [40, 41].

This is one of the reasons why knowledge transfer, and the co-production of foundational research with patient partners is so essential. Research has shown that while shared decision-making may not decrease the severity of the mental health disorder, it was reported to increase participation, treatment adherence and satisfaction with care amongst certain patient groups [42].

While the challenges can be intimidating, certain promising strides are being made to engage those with lived experience at both the foundational research and clinical levels. In Canada, the “Alberta Depression Research Priority Setting Project” asked clinicians and those with lived experience about the most important unanswered questions in depression research. Top priorities delineated include “Which treatment therapy or method is more successful for long term remission or recovery?”, “What are the long-term physical implications of pharmacotherapy for treating depression?”, and “For various treatment options … what are the advantages in terms of cost, effectiveness, relapse prevention and safety?”, alongside many others [28].

These priorities contrast with priorities listed without patient consultation, such as determining how traditional antidepressants work mechanistically and identifying underlying genetic factors behind this mental health disorder [43, 44]. While these are also essential avenues of research, it is imperative that foundational scientists realign our priorities to those of the patient. Incorporating patient opinions on a wider scale, including amongst communities with cultural differences who may outline different priorities, would be invaluable in setting research priorities for the next generation of mental health research. Please refer to Kirmayer, 2001 [45] for in-depth information on potential cultural differences in the experience, response to, treatment responsiveness, and side effect profiles of depression.

The lack of patient engagement in laboratory-based foundational research is evident. While clinicians have begun to create opportunities for patients to collaborate on their treatment plans and decision-making, PWLE are often left out of the beginning steps of mental health research. This, alongside the larger gaps in translation of terminology and understanding of scientific concepts, discourages participation in POR. This solidifies the need for a framework of engagement of PWLE in foundational research.

## Experiences incorporating POR into foundational science

### Patient perspectives

One of our co-authors first joined us in early 2018 via the BC SUPPORT Unit (British Columbia SUpport for People and Patient-Oriented Research and Trials), Vancouver Island Regional Centre. Her psychiatrist contacted our lead researcher thinking his patient would be a good fit for a CIHR grant application. Although we did not get the grant, this started a very meaningful relationship which continues to this day. Her work has spanned the spectrum of the research cycle including identifying study and research questions, understanding the lab processes, review of results, and knowledge translation. In her own words, “Being a patient partner is part of my personal therapy to stay well after years of treatment resistant depression. The best thing about being a patient partner is to take the mystery out of mental illness, and that has been pivotal to maintaining my wellness.” She has been instrumental in promoting our work via social media and at conferences and throughout our Health Authority, and has been the co-lead in a successful grant application to bring together all stakeholders in mental health patient-oriented research in our Health Authority. While opening the door for other patient partners, she has continually challenged us in positive ways to keep our research relevant to patients.

Another co-author first heard about POR at the 22nd Annual Vancouver Island Psychosis Conference, where she was attending as someone with lived experience. In talking with her, she cited many difficulties in having her opinion heard about her ongoing feelings of depression, anxiety and psychosis in a clinical setting, such as effectively communicating with her psychiatrist when her medication is not helping. She is in search of a different answer, or a more effective treatment plan that her clinician is unable to provide answers towards. Another issue is time. Often, psychiatrist appointments are rushed and do not allow time for patients to ask questions they may have about why or what they are receiving to combat their symptoms.

She states that: “the largest challenge so far in POR involvement was simply hearing about it, so I must stress the importance of advertising options available to patient groups directly to patients, not just interested academics.” Though she has only recently been involved in this format of research, she reported optimism and excitement in the current research being conducted, as well learning a lot about foundational research. She is looking forward to the next steps in her journey throughout POR.

### Student perspectives

While these collaborations have been at a beginning stage, interactions with those with lived experience has provided greater motivation to us, with many citing personal accounts from patients being a driving force of increased time and thoroughness spent on their work. Another benefit that we found was an increase in knowledge translation. Having patient partners attend student classes, seminars, and brainstorming sessions has increased the audience base that students can speak to and has made them all deeply consider the applicability of their research to real-world situations. At the University of Victoria, patient partners have spoken at a graduate course for translational neuroscience, and attended a Ted-Talks-like seminar series, where they posed questions to student presenters from various labs regarding their research interests. This includes considerations such as cost, accessibility, and feasibility of treatment plans. In one case, a student is now evaluating the efficacy of a pharmacological treatment for depression in the periphery to determine the best method that will still work to avoid patient discontinuation of medications. Our patient partners will be engaged throughout this project, including sharing their expertise towards side effect profiles, feasibility of introducing the intervention to their lifestyles, and preferences they have heard from their surrounding community. The addition of biological data to practical applications will hopefully allow us to co-produce guidelines in the future on the clinical use and implications of these biomarkers.

Patient-oriented research has provided new approaches and perspectives to all of the graduate students involved in our lab. It is often easy to get lost in specific aspects of our research and lose sight of the big picture. When patients share their experiences and questions, there is a reminder of the multifaceted considerations and priorities we need to have as researchers. Our patient partners have been mentors, guiding us through their lived experiences and issues they have faced inside and outside the medical community, particularly as those who have lived experience with mental health disorders.

### Principal investigator experiences

For the past 25 years the Principal Investigator (PI), one of the co-authors, has been actively working on the neurobiology of mood and psychotic disorders. In the past, his major efforts were devoted to foundational studies on animal models or human postmortem brain samples, but for the past decade he has also been involved in studiers in the clinical setting, and for the last three years he has incorporated a POR component to his research. A long time ago, while working in a Psychiatric Institute within a Psychiatric Hospital, he had the daily opportunity to meet and have a chat with patients. The insights that they could provide in relation to current research was evident. Their curiosity and keenness to provide ideas and to explain “what they needed” was invaluable. That vision was reinforced in conversations with close family who suffered from mental illnesses. However, it was not until 2017 when the author had the opportunity through the BC SUPPORT Unit to develop the proper contacts, explain his experiences, and bring forward the possibility that patient partners with mental disorder could be engaged in foundational science research. Conceptually this presented some advantages, as our lab was interested in tackling some issues related to the discovery of novel, faster, and more efficient antidepressant drugs. This was a line of research that patient partners deemed as extremely important and thereby were quite keen to try to find a way to incorporate their experiences onto this line of research. This included indicating what symptoms, according to their experience, were critical to tackle by antidepressant drugs, as well as what expected therapeutic outcomes they would deem as acceptable. However, our lab also knew that foundational research in animal models was far away from the knowledge and experiences of our patient partners and were aware that POR usually takes place in the clinical or public/health settings; not within foundational science laboratories. Thereby, our lab was both hopeful and a little bit skeptical of the possibilities to incorporate a POR component to our investigations.

After two and a half years of working directly with patient partners on mental health, the PI can assert that POR is not only possible within foundational science laboratories, but it has had a big effect in enriching the activities, training and knowledge of all the members of our laboratory (both students and PIs), however there needs to be a series of steps and recommendations that should be followed to foster POR in foundational mental health research.

### Patient-oriented research advisory committee experiences

Our Patient-Oriented Research Advisory Committee, funded through the BC SUPPORT Unit, is composed of six PWLE, two researchers, two clinicians, and one administrative decision-maker involved in mental health and POR. The scope of these meetings involves two series of discussions (one surrounding mood disorders, and the other surrounding psychotic disorders) with two meetings each (one directed by a PWLE, the other directed by a researcher). Through this, we hope to gain insight into the varying experiences of everyone who participates in the mental health system, and how we may better approach multidisciplinary mental health care. Due to COVID-19, in-person meetings have been delayed, however we have begun online meetings with everyone involved. Recent questions have gravitated towards the experience of mental health in COVID-19, with main questions such as “How is COVID-19 impacting those already diagnosed with mental health disorders?”, “How has the mental health of those who have had/have COVID-19 been impacted?”, “How is the mental health of frontline workers been impacted, particularly in places with high positivity rates?”, and “How are special populations impacted (such as the elderly, or young adults)”. We hope to share the conclusions and insights from our meetings at a later date.

### Examples of practical outcomes of incorporating PWLE to our laboratory research

While in sections 2.1 to 2.3. we outlined the personal experiences of patient partners, students and principal investigator as result of our POR approach, it becomes essential to detail the practical outcomes for our research that were a direct result of such enterprise. As such, we would like to describe in detail three main activities, one pertaining to our human research, a second to animal research, and a third involving both human and animal research, that were directly influenced by our patient partners.

#### Biomarker studies of human depression

For the past decade, the principal investigator has been involved in studies of protein expression in blood lymphocytes to gain information regarding to therapeutic response to antidepressant medication. These potential biomarkers of therapeutic efficacy in depression will help identifying patients that will respond well of not to antidepressant drug treatment if properly validated [46–49]. One of our patient partners (and co-author of this manuscript) suffered from treatment resistant depression and was not responding properly to antidepressant drug therapy. However, she did respond well when subjected to repetitive transcranial magnetic stimulation, a first line therapy recommendation for patients that have failed at least one antidepressant drug trial, according to the Canadian Network for Mood and Anxiety Treatments [50]. She asked us about the possibility of using our biomarkers approach to evaluate the therapeutic response to rTMS (repetitive Transcranial Magnetic Stimulation) for treatment resistant depression patients, and she put us in contact with a psychiatrist in British Columbia (BC) who is a prominent researcher in the field. As a consequence, we are currently finishing recruiting patients to complete a study on the changes in protein expression on peripheral lymphocytes before and after rTMS treatment. Although we do not have the data finalized yet (as we get coded blood samples for our analyses and the code will not be broken until the end of recruitment and treatment), we would not have engaged in this study if not by the direct engagement of our patient partner, who not only indicated the possible interest of such a study but also provided the avenue for us to be able to do it.

#### Behavioral studies on animal models

An important component of our research is studies on animal models that tend to mimic some aspects of interest of mental disorders. While there are numerous limitations in the use of animals to model specific aspects of mood disorders, there are different paradigms that allow the study of behavioral disturbances that resemble some components of depression in humans. For a long time, we have been using paradigms based on giving the animals daily doses of the stress hormone corticosterone [51, 52]. The administration of corticosterone results in depressive-like behavior that can be ascertained in a laboratory setting and allows preclinical studies of antidepressant drugs. The most commonly analyzed behavior is the “forced-swim test”, which has been used since the 1970s to evaluate despair and the effectiveness of antidepressant drugs. Animals are taken to a container with water and they actively swim or are immobile with only their nose out of the water, which is considered a measure of giving up (despair) and is induced by chronic stress and reversed by antidepressants. The daily administration of stress hormones results in an increase of the immobility of rodents in the forced-swim test, mimicking the despair observed in human patients. Our patient partner was quite impressed with this, but she was mentioning that there were other depression symptoms that we perhaps should evaluate in our models if possible, like anhedonia (the inability to enjoy experiences that we consider quite pleasurable) or cognitive symptoms and memory. We believed this of interest and thereby are increasing the repertoire of behavioral tasks evaluated and have recently published a paper in which we evaluated cognition in our animal model studies designed to investigate putative novel antidepressants [53].

#### Studies on psychosis

Around 20 years ago the principal investigator was involved in research demonstrating that brains of those diagnosed with schizophrenia show about half levels of the extracellular matrix protein Reelin [54]. Since then, we have primarily been evaluating this protein in depression (where Reelin is primarily decreased in areas involved in emotion and memories), but have also proposed an animal model that could be useful to study aspects related to schizophrenia at the prodromal stage [55]. Our recent interactions with PWLE with psychosis have rekindled our interests in these studies at the prodromal onset and at the onset of psychosis symptoms (an aspect that both people with lived experience and mental health practitioners consider of great interest). Thereby, prompted by our patient partner and also by people from our “Advisory Committee for POR in Mental Health” (see Section 3.3) we have contacted the head of the Canadian Consortium for Early Intervention in Psychosis to evaluate the possibility of partnering in the continuation of our studies on schizophrenia prodrome and onset. This is a new avenue that we are just starting from a direct consequence of our interactions with patient partners.

In summary, keeping an open mind in conversations with PWLE who have partnered with us in the lab setting has definitely helped us to develop novel ideas and open new avenues of research.

### COVID-19 and the continuation of POR

COVID-19 (Coronavirus disease) has had unique advantages and disadvantages for the incorporation of POR into foundational science. With many lockdowns, access to the lab has been severely restricted, preventing patient partners from having direct access to researchers, as well as stalling foundational science in general. While email and zoom can provide a proxy for patient-researcher contact, this seems to be more of a maintenance stage of the relationship than actual progression towards generating future avenues. Lastly, recruitment of new patient partners has stopped completely, as the importance of social distancing does not allow for the introduction of new people to the research group.

For our patient partners, COVID-19 has over-shadowed POR and in some cases stopped other patient engagement opportunities outright. However, based on the established relationships with the lab some new opportunities arose, including this paper. The proven strategy for POR in mental health, that is open and transparent communication (via Zoom and the internet) has bridged the gap between patient partners and the PI and the students.

As mentioned, COVID-19 has created new formatting and reliance on technologies such as Zoom, which allows for broader communication and participation in POR that is not limited by geographical area. Less time spent in the lab has also led to greater participation in online discussions, conferences, and more as well as the improvement of students’ own online presences for sharing their work to a large audience. As everyone navigates further through this pandemic, patient partners and researchers will hopefully generate innovative methods to foster POR, improving these connections far into the future.

## Recommendations for incorporating POR into foundational science

Please keep in mind that these recommendations primarily reflect the incorporation of POR in the context of Canada, particularly in BC, as our patient partners and researchers share their experiences from within that administrative framework. They are based off the experiences of our research group and advisory committee (composed of 2 clinicians, 1 administrator, 6 people with lived experience, and 2 basic researchers), and we understand many labs will have varying experiences. The degree of involvement and engagement of each person with lived experience will need to be evaluated at an individual level, so please take caution in the generalization of these guidelines.

### Initial recruitment

The first step is advertisement, and greater dissemination of information on POR. As mentioned previously in our experiences, it is difficult for some patients to know that they could be involved in this type of research. Advertisements could take place through local clinicians that inform their patients about these forms of opportunities, or through conferences on mental health that are open to patients and their support systems. These conferences, such as the 22nd Annual Vancouver Island Psychosis Conference, have been effective at gathering initial participation interest from varied patient groups.

After initial contact is made with the research team, we have found that having an established patient partner working with the research team “meet and greet” a new patient partner builds trust and provides a secure safe place to talk about the research. Usually the meeting takes place away from the lab, in a neutral setting like a coffee shop. This aids in avoiding potential barriers to effective patient-oriented research, such as behaviors and settings that enforce differential power dynamics.

### Building the partnership

After the initial meeting with the established patient partner, the new patient partner is introduced to the team either one-on-one (with the principal investigator) or as a group (for instance, students working in the lab). A family member may be present during these meetings if preferred by the patient partner, to help build the support system and educate not only the patient, but those who care for them.

We found that once the new patient partner feels comfortable with one person, the relationship will be co-built, with each person bringing new ideas to the research. One of our patient partners presented a graduate seminar on her mental health journey. That same patient partner attended a tutorial for graduate students to review their work from her lens of lived experience with treatment resistant depression.

Co-building the relationship between the patient partners and researchers takes time and effort from both the patients and researchers. In order to facilitate communication, the University of Victoria hosts a translational neuroscience course that allows students to interact with patients and members of patient organizations in a more casual setting, where group discussion with the patients as a leader and students sharing their research allowed the development of a shared and equal language. Training and discussion such as this should be implemented in many graduate programs, as earlier training allows a span of graduate students to consider and incorporate POR into their studies.

### Conducting POR in foundational science

Early on, patient partners should indicate the amount of involvement that they feel comfortable with, dependent on many individual factors such as availability, social comfort, and more. Concerns of privacy can also be an issue, and patient partners may not want to be identified due to potential stigma. On the other hand, certain partners value sharing their experiences with larger audiences, such as two of our patient partners who have co-authored papers and make regular presentations. Consistent discussions regarding each patient partners’ preferences should circumvent any issues. There are also certain restrictions to consider depending on the research setting, as some labs have rules in place regarding who can enter certain areas. In labs where direct observation of the research is not possible, researchers should find a neutral area to discuss their current ideas and results, where patient feedback can still have a direct impact on the foundational science occurring inside of the lab.

For our own lab, we face a unique issue in that we conduct animal research. This is a sensitive topic for many people, and it is important to establish the level of comfort patient partners have in engaging in these forms of research. The patient partner co-authors cite the importance of information surrounding animal use ethics, as well as the direct attempts of researchers to minimize suffering and number of animals used. Understanding that the use of animals was necessary for developing novel treatments and biomarkers also helped put this into perspective. One co-author emphasizes that the researchers should keep up to date with potential substitutions for animal use, and design research questions accordingly. These types of conversations are what made them feel most comfortable in an animal-use lab. If a patient is not comfortable with any form of animal use, involvement in other lab projects is still possible. For example, our involvement in blood biomarkers for treatment-responsiveness in human patients would be appropriate. It is important to establish boundaries and guidelines for each individual patient partner.

After the first few meetings, and depending on availability of all parties involved, direct contact may begin to fade. Patient partners need to know the research team is receptive to new ideas. They will interact with the team in different ways, through e-mail, phone or (pre-COVID-19) coming into the lab to discuss results. Contact information for all members involved should be shared and readily available to all. Patient partners are curious and willing to learn – and most important, they need the knowledge that their ideas will be considered and discussed. As part of this, we recommend forming an advisory committee on the development of POR in laboratory and mental health research, including the patient partners who wish to be involved, researchers and students, clinicians, and healthcare workers. We have found that regular interactions of the committee (around once every one to two months) with focused topics (such as depression and psychosis in our situation) and ample time for discussion lead to increased comfort, more ideas from different perspectives, and the assurance of patient partners being integral to the decision-making process.

Another function of a POR advisory committee is to determine compensation for patient partners. Compensation is often essential, as we ask for time and labour from the patient partners to contribute to our research and policy going forward. Research has denoted five important reasons to compensate patient partners: equity, different motivations than researchers, respect for vulnerability, commitment, and barrier removal [39, 40]. For a detailed roadmap on patient compensation in Canada, contributed to by researchers and patient partners, Richards and colleagues have provided an invaluable resource [56, 57]. While we do not have a template yet for our local area, this has been a topic of conversation in our advisory panel to determine the appropriate amount of compensation for patient partners.

To ensure the consideration of feedback, foundational researchers could write up a short background and brief experimental design to share and discuss with patient partners. This would be helpful on both sides, as researchers could address any subconscious biases by cementing the proposed research in their minds and gathering any previous quantitative or qualitative data on the subject at hand. For patient partners, this allows them to see both the qualities and potential limitations of foundational science in addressing their concerns. As these discussions continue, researchers and patients will get closer to communicating on similar levels, adjusting to better fit with the other person in order to create a fruitful and productive workspace together.

It is important to remember that good things take time. Our team has gradually built up the relationship with patient partners, attending local conferences together, going for coffee, and always being open and communicative with any questions. The stronger the relationships are, and the more experience everyone has in being involved with POR in foundational science, the easier it is to incorporate further people on both sides. We hope with our experiences and knowledge in this field we can continue to increase patient involvement and outreach, and have our patient partners educate other researchers and patient partners on what it means to truly be involved in patient-oriented research.

## Conclusions

The necessity to prioritize active partnerships between patients and researchers are evident. By providing direct access to foundational scientists, the voices of patient partners shape research towards translational outcomes that greater benefit the patient population. This is especially important in mental health research, where the voices of those with lived experience are often downplayed or discredited. The exchange of knowledge between patients and researchers are advantageous on both sides. For patients, this can enhance confidence when approaching their healthcare practitioners or perhaps increase optimism in the work that is being conducted. For researchers, this generates ideas and input that actually benefit the group they are modeling, increases motivation towards their research, and provides a greater understanding of how this impacts individual patients. The fostering of a close relationship between patients and foundational researchers is essential to improving the future of translational science, mental health research, and patient-oriented research.

## Data Availability

Not applicable.

## Abbreviations

BC: British Columbia
BC SUPPORT Unit: British Columbia Support for People and Patient-Oriented Research and Trials Unit
CIHR: Canadian Institute of Health Research
IAP2: International Association for Public Participation 2
JLA: James Lind Alliance
LGBTQIA2S: Lesbian, Gay, Bisexual, Transgender, Queer and/or Questioning, Intersex, Asexual, Two-Spirit, plus the countless ways in which people choose to self-identify
NIHR: National Institute of Health Research
PI: Principal Investigator
POR: Patient-Oriented Research
PSP: Priority Setting Partnerships
PWLE: People with Lived Experience
rTMS: repetitive Transcranial Magnetic Stimulation
SPOR: Strategy for Patient-Oriented Research
STEM: Science, Technology, Engineering, and Mathematics
UK: United Kingdom

## References

[1] Krahn M, Naglie G (2008). The Next Step in Guideline Development: Incorporating Patient Preferences. JAMA.

[2] Schünemann HJ, Fretheim A, Oxman AD (2006). Improving the use of research evidence in guideline development: 10. Integrating values and consumer involvement. Heal Res policy Syst.

[3] Elberse JE, Caron-Flinterman JF, JEW B (2011). Patient–expert partnerships in research: how to stimulate inclusion of patient perspectives. Heal Expect.

[4] UK Standards for Public Involvement. The UK Standards. [cited 2021 Mar 3]. Available from: https://sites.google.com/nihr.ac.uk/pi-standards/standards

[5] Canadian Institute of Health Research (2014). Foundations of SPOR [Internet]. Strategies for SPOR.

[6] Hickey G, Brearley S, Coldham T, Denegri S, Green G, Staniszewska S, Tembo D, Torok K, Turner K (2018). Guidance on co-producing a research project.

[7] Vogel R, Moulder E, Huggins M (2014). The extent of public participation. Public Manag.

[8] Sacristán JA (2013). Patient-centered medicine and patient-oriented research: improving health outcomes for individual patients. BMC Med Inform Decis Mak.

[9] Caron-Flinterman JF, Broerse JEW, Bunders JFG (2005). The experiential knowledge of patients: a new resource for biomedical research?. Soc Sci Med.

[10] Williamson C (2008). The patient movement as an emancipation movement. Heal Expect Int J Public Particip Heal Care Heal Policy.

[11] Boaz A, Biri D, McKevitt C (2016). Rethinking the relationship between science and society: has there been a shift in attitudes to Patient and public involvement and public engagement in science in the United Kingdom?. Heal Expect an Int J public Particip Heal care Heal policy..

[12] Caron S (2020). Addressing the disparities in health care and health outcomes between Indigenous and non-Indigenous peoples in Canada.

[13] Buchmueller TC, Levy HG (2020). The ACA’s Impact On Racial And Ethnic Disparities In Health Insurance Coverage And Access To Care: An examination of how the insurance coverage expansions of the Affordable Care Act have affected disparities related to race and ethnicity. Health Affairs.

[14] Universities of Canada (2019). Equity, diversity, and inclusion at Canadian Universities Canada.

[15] Owens A, Holroyd BR, McLane P (2020). Patient race, ethnicity, and care in the emergency department: a scoping review. Can J Emerg Med.

[16] Tobon AL, Flores JM, Taylor JH, Johnson I, Landeros-Weisenberger A, Aboiralor O, Bloch MH. Racial Implicit Associations in Psychiatric Diagnosis, Treatment, and Compliance Expectations. Acad Psychiat. 2021;45(1):23–33. https://link.springer.com/article/10.1007/s40596-020-01370-2. 10.1007/s40596-020-01370-2 PMC7933096 33438155

[17] Universities of Canada (2019). Equity, diversity, and inclusion at Canadian Universities Canada.

[18] Employment Equity Survey (2018). University of Victoria.

[19] Liu SN, Brown SE, Sabat IE (2019). Patching the “leaky pipeline”: Interventions for women of color faculty in STEM academia. Archives of Scientific Psychology.

[20] Johnston JN, Thacker JS, Desjardins C, Kulyk BD, Romay-Tallon R, Kalynchuk LE, Caruncho HJ (2020). Ketamine Rescues Hippocampal Reelin Expression and Synaptic Markers in the Repeated-Corticosterone Chronic Stress Paradigm. Front Pharmacol.

[21] Caruncho HJ, Rivera-Baltanas T, Romay-Tallon R, Kalynchuk LE, Olivares JM (2019). Patterns of Membrane Protein Clustering in Peripheral Lymphocytes as Predictors of Therapeutic Outcomes in Major Depressive Disorder. Frontiers in pharmacology.

[22] NIH National Center for Advancing Translational Sciences. What is Translational Science [Internet]. Available from: https://www.tuftsctsi.org/about-us/what-is-translational-science/. Accessed 6 June 2020.

[23] Butler D (2008). Translational research: crossing the valley of death. Nature.

[24] Eisenmann J (2017). Translational gap between laboratory and playing field: new era to solve old problems in sports science. Transl J Am College Sports Med.

[25] Tkacs NC, Thompson HJ. From bedside to bench and back again: research issues in animal models of human disease. Biol Res Nursing. 2006;8(1):78–88.https://journals.sagepub.com/doi/abs/10.1177/1099800406289717?casa_token=_GuPj6AaLX0AAAAA:Jm4zY6jn57uLCY4rgJZTXK-6H0v5inSkjojtQQlg4CKCWO-DpbYiKhN19dvCcTDzDKCpGFOP0D_t0w. 10.1177/1099800406289717 PMC2366101 16766631

[26] Moore DR (2008). Reverse translation: clearing a path from bedside to bench. Nature.

[27] Fonseka TM, Pong JT, Kcomt A, Kennedy SH, Parikh SV (2019). Collaborating with individuals with lived experience to adapt CANMAT clinical depression guidelines into a patient treatment guide: The CHOICE-D co-design process. J Eval Clin Pract.

[28] Breault LJ, Rittenbach K, Hartle K, Babins-Wagner R, de Beaudrap C, Jasaui Y, Ardell E, Purdon SE, Michael A, Sullivan G, Unger A”SR, Vandall-Walker L, Necyk B, Krawec K, Manafò E, Mason-Lai P (2018). The top research questions asked by people with lived depression experience in Alberta: a survey. C Open.

[29] Grimshaw JM, Eccles MP, Lavis JN, Hill SJ, Squires JE. Knowledge translation of research findings. Implement Sci. 2012;7(1):50. Available from: 10.1186/1748-5908-7-50. 10.1186/1748-5908-7-50 PMC3462671 22651257

[30] James Lind Alliance. James Lind Alliance Priority Setting Partnerships. Available from: https://www.jla.nihr.ac.uk/about-the-james-lind-alliance/about-10.1186/s40900-021-00266-1psps.htm. Accessed 14 Dec 2020.

[31] Lophatananon A, Tyndale-Biscoe S, Malcolm E, Rippon HJ, Holmes K, Firkins LAm, Fenton M, Crowe S, Stewart-Brown S, Gnanapragasam VJ, Muir KR The James Lind Alliance approach to priority setting for prostate cancer research: an integrative methodology based on patient and clinician participation. BJU Int 2011;108(7):1040–1043, DOI: 10.1111/j.1464-410X.2011.10609.x. 10.1111/j.1464-410X.2011.10609.x 21914107

[32] Madden M, Morley R (2016). Exploring the challenge of health research priority setting in partnership: reflections on the methodology used by the James Lind Alliance pressure ulcer priority setting partnership. Res Involv Engagem.

[33] Knight SR, Metcalfe L, O’Donoghue K, Ball ST, Beale A, Beale W, Hilton R, Hodkinson K, Lipkin GW, Loud F, Marson LP, Morris PJ (2016). Defining priorities for future research: results of the UK kidney transplant priority setting partnership. PLoS One.

[34] Manns B, Hemmelgarn B, Lillie E, Dip SCPG, Cyr A, Gladish M, Large C, Silverman H, Toth B, Wolfs W, Laupacis A (2014). Setting research priorities for patients on or nearing dialysis. Clin J Am Soc Nephrol.

[35] Kelly S, Lafortune L, Hart N, Cowan K, Fenton M, Brayne C (2015). Dementia priority setting partnership with the James Lind Alliance: using patient and public involvement and the evidence base to inform the research agenda. Age Ageing.

[36] Hollis C, Sampson S, Simons L, Davies EB, Churchill R, Betton V, Butler D, Chapman K, Easton K, Gronlund TA, Kabir T (2018). Identifying research priorities for digital technology in mental health care: results of the James Lind Alliance Priority Setting Partnership. Lancet Psychiatry.

[37] Morris C, Simkiss D, Busk M, Morris M, Allard A, Denness J, Cowan K. Setting research priorities to improve the health of children and young people with neurodisability: a British Academy of Childhood Disability-James Lind Alliance Research Priority Setting Partnership. BMJ open. 2015;5(1). https://bmjopen.bmj.com/content/5/1/e006233.short. 10.1136/bmjopen-2014-006233 PMC4316435 25631309

[38] Prior M, Bagness C, Brewin J, Coomarasamy A, Easthope L, Hepworth-Jones B, Hinshaw K, O'Toole E, Orford J, Regan L, Raine-Fenning N (2017). Priorities for research in miscarriage: a priority setting partnership between people affected by miscarriage and professionals following the James Lind Alliance methodology. BMJ Open.

[39] Breault LJ, Rittenbach K, Hartle K, Babins-Wagner R, de Beaudrap C, Jasaui Y, Ardell E, Purdon SE, Michael A, Sullivan G, Vandall-Walker L (2018). People with lived experience (PWLE) of depression: describing and reflecting on an explicit patient engagement process within depression research priority setting in Alberta, Canada. Res Involvement Engagement.

[40] Hamann J, Cohen R, Leucht S, Busch R, Kissling W (2007). Shared decision making and long-term outcome in schizophrenia treatment. J Clin Psychiatry.

[41] Hamann J, Langer B, Winkler V, Busch R, Cohen R, Leucht S, Kissling W (2006). Shared decision making for in-patients with schizophrenia. Acta Psychiatr Scand.

[42] Duncan E, Best C, Hagen S (2010). Shared decision making interventions for people with mental health conditions. Cochrane Database Syst Rev.

[43] Nestler EJ, Gould E, Manji H (2002). Preclinical models: status of basic research in depression. Biol Psychiatry.

[44] Insel TR, Charney DS (2003). Research on Major DepressionStrategies and Priorities. JAMA.

[45] Kirmayer LJ (2001). Cultural variations in the clinical presentation of depression and anxiety: implications for diagnosis and treatment. J Clin Psychiatry.

[46] Rivera-Baltanas T, Olivares JM, Calado-Otero M, Kalynchuk LE, Martinez-Villamarin JR, Caruncho HJ (2012). Serotonin transporter clustering in blood lymphocytes as a putative biomarker of therapeutic efficacy in major depressive disorder. J Affect Disord.

[47] Rivera-Baltanas T, Olivares JM, Martinez-Villamarin JR, Fenton EY, Kalynchuk LE, Caruncho HJ (2014). Serotonin 2A receptor clustering in peripheral lymphocytes is altered in major depression and may be a biomarker of therapeutic efficacy. J Affect Disord.

[48] Rivera-Baltanas T, Agis-Balboa RC, Romay-Tallon R, Kalynchuk LE, Olivares JM, Caruncho HJ (2015). Serotonin transporter clustering in blood lymphocytes predicts the outcome on anhedonia scores in naïve depressive patients treated with antidepressant medication. Ann General Psychiatry.

[49] Caruncho HJ, Rivera-Baltanas T, Romay-Tallon R, Kalynchuk LE, Olivares JM (2019). Patterns of membrane protein clustering in peripheral lymphocytes as predictors of therapeutic outcomes in major depressive disorder. Front Pharmacol.

[50] Milev RV, Giacobbe P, Kennedy SH, Blumberger DM, Daskalakis ZJ, Downar J, Modirrousta M, Patry S, Vila-Rodriguez F, Lam RW, MacQueen GM, Parikh SV, Ravindran AV (2016). CANMAT depression work group. Canadian network for mood and anxiety treatments (CANMAT) 2016 clinical guidelines for the Management of Adults with major depressive disorder: section 4. Neurostimulation treatments. Can J Psychiatr.

[51] Sterner EY, Kalynchuk LE (2010). Behavioral and neurobiological consequences of prolonged glucocorticoid exposure in rats: relevance to depression. Prog Neuro-Psychopharmacol Biol Psychiatry.

[52] Fenton EY, Fournier NM, Lussier AL, Romay-Tallon R, Caruncho HJ, Kalynchuk LE (2015). Imipramine protects against the deleterious effects of chronic corticosterone on depression-like behavior, hippocampal reelin expression, and neuronal maturation. Prog Neuro-Psychopharmacol Biol Psychiatry.

[53] Brymer KJ, Johnston J, Botterill JJ, Romay-Tallon R, Mitchell MA, Allen J, Pinna G, Caruncho HJ, Kalynchuk LE (2020). Fast-acting antidepressant-like effects of Reelin evaluated in the repeated-corticosterone chronic stress paradigm. Neuropsychopharmacology..

[54] Impagnatiello F, Guidotti AR, Pesold C, Dwivedi Y, Caruncho H, Pisu MG, Uzunov DP, Smalheiser NR, Davis JM, Pandey GN, Pappas GD, Tueting P, Sharma RP, Costa E (1998). A decrease of reelin expression as a putative vulnerability factor in schizophrenia. Proc Natl Acad Sci U S A.

[55] Lussier AL, Romay-Tallón R, Kalynchuk LE, Caruncho HJ (2011). Reelin as a putative vulnerability factor for depression: examining the depressogenic effects of repeated corticosterone in heterozygous reeler mice. Neuropharmacology..

[56] Richards DP, Jordan I, Strain K, Press Z (2018). Patient partner compensation in research and health care: the patient perspective on why and how. Patient Exp J.

[57] Richards DP, Jordan I, Strain K, Press Z (2020). Patients as partners in research: how to talk about compensation with patient partners. J Orthop Sport Phys Ther.

